# A mixed-methods analysis of a do-it-yourself e-cigarette community on Reddit

**DOI:** 10.18332/tpc/211846

**Published:** 2025-12-11

**Authors:** Arpita Tripathi, Kar-Hai Chu

**Affiliations:** 1 Department of Behavior and Community Health Sciences, School of Public Health, University of Pittsburgh, Pittsburgh, United States

**Keywords:** social media, flavor ban, e-cigarettes, nicotine and tobacco policy, DIY methods

## Abstract

**INTRODUCTION:**

Flavored e-cigarette use among US youth remains prevalent, prompting regulatory action by the Food and Drug Administration (FDA). On 2 January 2020, the FDA announced a federal ban on flavored e-cigarettes, which may be circumvented through unregulated do-it-yourself (DIY) methods shared in online communities. Understanding discourse within these communities is essential to understanding unintended policy effects. This study’s primary aim was to describe discourse in a DIY e-cigarette subreddit, with a secondary aim of examining how discussions shifted following the 2020 flavor ban.

**METHODS:**

We conducted a mixed-methods study of posts from the subreddit r/DIY_eJuice, an online community focused on DIY e-liquid mixing. A total of 5110 posts were extracted between January 2019 and January 2021, with posts before 2 January 2020 defined as the pre-ban period and those after as the post-ban period. From this dataset, we randomly selected 800 posts (17% of the total; 400 pre-ban and 400 post-ban), which were coded by three trained human coders using a systematically developed codebook with 12 thematic categories. Chi-squared tests were applied to compare thematic distributions between periods.

**RESULTS:**

DIY mixing methods (76%, n=605) and discussions about flavors (49%, n=390) were the most frequent topics across both periods, despite the flavor ban. Policy-related discussions significantly increased from 3.5% (n=14) pre-ban to 8.3% (n=33) post-ban (p=0.004). Posts related to safety concerns remained infrequent in both pre- and post-ban period. The proportion of beginner users was constant at 22% across both periods (n=90 at pre-ban, and n=88 at post-ban), while discussions by experienced users increased from 35.3% (n=141) to 41.5% (n=166).

**CONCLUSIONS:**

Regulatory action on flavored e-cigarettes influenced community discourse, increasing conversations about policy and DIY mixing techniques, but not significantly affecting discussions of safety. Continued surveillance of DIY communities is necessary to inform future public health strategies.

## INTRODUCTION

The growing popularity of electronic cigarettes, or e-cigarettes, especially amongst adolescents and young adults remains a continued public health concern^1^. E-cigarette use has been associated with various health conditions including respiratory disorders^2^, cognitive disorders^3^, cardiovascular risks^4^, and potential nicotine addiction^5^. Additionally, there is evidence that e-cigarette use can be a pathway to future cigarette smoking^6^. Moreover, surveys with adolescents have reported that perceived harm of e-cigarettes is lower amongst e-cigarette users than amongst non-users^7^.

It is estimated that in 2023, around 2.1 million (7.7%) high-school and middle-school students used e-cigarettes in the past 30 days^8^. E-cigarette flavors make them highly appealing to the youth, masking the traditionally strong and pungent flavors of cigarettes^9^. In 2019, more than 66% of the users reported that they used e-cigarettes because of the variety of available flavor^10^, while in 2023, nearly 89% of the youth reported using flavored e-cigarettes^8^.

In an effort to limit e-cigarette use and initiation, policy-makers implemented various regulatory mechanisms to protect young people from accessing flavored e-cigarettes. For example, between 2019 and 2020, the states of New York, New Jersey, Massachusetts, and Rhode Island introduced bans on the sale of flavored e-cigarettes and flavored e-liquids^11,12^. Further, on 2 January 2020, the Food and Drug Administration (FDA) announced a federal ban on the sale of all flavored e-cigarettes cartridges except menthol and tobacco flavor^13^.

These bans, however, can be circumvented by the under-researched do-it-yourself (DIY) methods for making e-cigarette liquids, or e-juice, by combining commonly available ingredients. For example, instructions available online to make DIY e-cigarette liquids include mixing a base liquid of propylene glycol (PG) and vegetable glycerin (VG), nicotine, and flavor concentrates^14^. The individual ingredients for DIY methods, such as mixing bottles, measuring syringes etc. are widely available and can be purchased from shops and/or online retailers^15^.

Researchers have expressed concern about the safety of the practice^16,17^, while its prevalence remains unknown. A previous study explored the motivations of individuals that used DIY e-cigarette methods^18^. The authors interviewed 41 participants from the UK to examine their motivations to use DIY e-cigarette methods and analyzed the chemical composition of the e-cigarette liquids created by the participants. They concluded that the major reason to use DIY methods included fun/novelty of the practice, desire to achieve higher nicotine levels than those in commercially available liquids, reduction in cost, and ability to control quality of the liquids. In addition, they also found that 66% of their participants first heard about DIY e-liquid methods on online communities. Further, another study conducted with international participants reported that youth that perceived e-cigarettes to be safer were more likely to experiment with DIY e-cigarette mixing methods^19^.

Social media communities have been linked to a rise in the online sales of unregulated substances^20^. Due to the lack of data on the prevalence of DIY e-cigarette methods, it has been challenging to examine this issue comprehensively^19^. The current study presents a systematic approach to analyze the discourse within online communities that use DIY e-cigarette methods, aiming to assess the impact of federal flavor ban on the DIY e-cigarette user community. More broadly, this analysis is important for understanding the unintended and unexpected consequences of flavor bans in general. To the best of our knowledge, there currently is no known work in the US that examines the prevalence or popularity of DIY vaping methods on online forums.

Reddit, a popular social media platform organized into topic-specific ‘subreddits’, provides a diverse and dynamic environment for collecting data and gaining insights into user behavior, opinions, and trends not generally available in traditional surveillance methods^21^. The platform has been used for exploring a myriad of emerging public health issues including vaccine acceptance and skepticism^22^, substance use and addiction science^23^.

As of 2023, Reddit was the 10th most popular website in the US^24^. Approximately 49% of Reddit users are from the US^25^, making it a valuable platform to understand health behaviors ^26^. Demographically, 36% of US adults aged 18–29 years and 22% of those aged 30–49 years are Reddit users^25^, supporting its relevance for studying young adult populations that are central to e-cigarette use.

Within Reddit, the DIY_eJuice subreddit is the largest and most active community that discusses DIY e-liquids. The subreddit has been active since 2021, and had approximately 74000 members as of 2023. This community offers a unique lens into the practices, motivations, and experiences of individuals engaged in DIY e-cigarette mixing.

This study is guided by two research questions. The primary aim is to describe the current discourse within DIY vaping communities on Reddit, a popular User Generated Content (UGC) platform. A secondary aim is to examine how this discourse shifted following the 2020 federal ban on flavored e-cigarettes, in order to explore potential changes in community discussions in response to the policy.

## METHODS

### Study design

This is a mixed-method study for understanding the current discourse and the impact of the 2020 federal flavor ban in the DIY vaping communities on the social media platform, Reddit. The study uses qualitative analysis to identify and categorize the discourse within the DIY communities and statistical methods to assess the impact of the flavor ban in the same community.

### Data collection

Following FDA’s announcement of the federal ban on flavored e-cigarette on 2 January 2020^13^, we extracted all posts from the subreddit DIY_eJuice between 1 January 2019 and 2 January 2021 to compare the discourse one-year before and one-year after the policy announcement. For analysis, we defined the pre-ban period between 1 January 2019 and 1 January 2020, and the post-ban period as 2 January 2020 and 2 January 2021. All posts on 2 January – which was the date of the FDA announcement – were included in the post-ban period to capture responses from the time of policy enforcement. Further, 2 January 2021 was included in our dataset to ensure a one-year comparison. We used the Python Reddit API Wrapper (PRAW), a library that allows access to Reddit’s Application Programming Interface (API) in order to collect publicly available data. The extraction yielded 5110 unique posts that we stratified between pre-ban (n=2742) and post-ban (n=2368) periods. This study was approved by the University of Pittsburgh Human Research Protection Office.

### Codebook development and coding procedure

To address the primary research question, the lead author (AT) developed an initial codebook for human coding based on prior social media research^27-31^ and a review of 100 random posts (2% of the dataset) from the DIY_eJuice subreddit. Following this, two research assistants with prior coding experience of social media content, independently applied the codebook to 25 random posts and double coded them. All discussions and disagreements regarding definitions of codes and their interpretation were resolved through an iterative adjudication process led by the lead author.

The final codebook was applied to a random subsample of 17% of the dataset (n=800 of 5110 posts; 400 from the pre-ban and 400 from the post-ban period). The 17% sampling fraction was calculated by dividing the number of coded posts (800) by the total dataset (5110). The stratification of pre-ban and post-ban posts was made equal to ensure balanced comparison across periods. The subsample size was chosen based on prior qualitative analyses of social media data^27,30^, which have typically analyzed between 10–20% of large datasets to balance resource constraints with thematic depth and comparative rigor. Research assistants worked independently to code 800 posts, and the lead author employed an iterative coding process that included resolving disagreements through weekly adjudication meetings. Coders were provided both the posts’ text, the username of the original authors of the post, and a corresponding link to the actual post online. All related posts that were publicly accessible during the coding phase were reviewed on the Reddit platform to enable evaluation of external content linked within the posts, whenever feasible.

Text originating from posts that were not accessible were still included in the thematic analysis to ensure the comprehensive nature of the original data was maintained. To ascertain inter-rater reliability, Cohen’s kappa coefficient was utilized, and any disparities between the coders were settled through adjudication, with the lead author (AT) holding the ultimate decision-making authority. Following five rounds of this iterative process, the inter-rater reliability was deemed to be excellent (Cohen’s κ values between 0.71 and 1.00) for all identified categories.

### Coding categories

Each post was coded based on the user’s experience with DIY methods, the intent of the post, and its thematic content. Full definitions for each code are available in Table 1.

**Table 1:** Example of final codebook used for content analysis

*Category*	*Code*	*Definition*	*Example*
**User experience**	Experienced user	Posts coded as ‘experienced’ if users indicated prior engagement with DIY e-liquid mixing (e.g. previously created recipes, used vendors/suppliers, reviewed flavors or techniques).	*‘Can anyone recommend a green apple flavor that is similar? Maybe someone already tried the MyBlu pods and know what I’m after. I want to avoid buying a lot of flavors again (since I already have around 100, but only use maybe like 30 of them [Face With Stuck-Out Tongue Emoji]).’* *‘Okay so a friend and I just made new e-juice but after steeping the flavor feels underwhelming and not as strong as expected. We used a Red Apple remix from DIYorDIE and we don’t know if something is wrong.’* *‘So I usually mix with freebase and accidentally ordered nicotine salt. I typically make a 60/40 vegetable glycerin (VG)/propylene glycol (PG) at 10% and make 5 mg juice.’*
	Beginner user	Posts coded as ‘beginner’ if users identified themselves as new to DIY mixing (e.g. used words like ‘first-timer’, ‘newbie’, ‘noob’)	*‘Looking into making DIY juices more and more given the current climate. Any tips to give to a first-timer?’* *‘So first time making DIY and I was wondering, why do people buy 1 liter vegetable glycerin (VG) and 1 liter propylene glycol (PG) then mix it to 70/30 when you can buy 1 liter 70/30 premade? I’m a noob to DIY so don’t roast me [Face With Tears of Joy Emoji].’*
**Post intent**	Technical question/seeking recommendation or advice	Posts asking technical, method, or process-based questions, or seeking recommendations on flavors, recipes, mixing methods, or troubleshooting.	*‘Overflowing e-liquid? I put in everything in the calculator trying to make 120 ml but it always overflows. Am I doing something wrong?’* *‘Have you all experienced this with the nicotine hit salt? Either way, the taste is very different than the smooth I’ve purchased in the past.’* *‘Will these flavors cut it for someone who smoked real tobacco? Can anyone recommend a green apple flavor that is similar?’*
	Sharing personal experience/giving advice/discussion	Posts narrating DIY experiences, reviewing products, or providing advice.	*‘Vampire Vape Grape tastes like plastic.’*
**Themes**	Flavor profile	Posts discussing or reviewing flavor combinations, requesting flavor recommendations, or commenting on flavor notes.	*‘German Flavors Enhanced Sweetener.’* *‘Hi there. Just wanted to make some juice and I’m wondering, can I use only one flavor or do I need two or more?’*
	Recipes and ingredients	Posts sharing or requesting recipes, discussing mixing processes, ingredient measurement, or nicotine salts.	*‘Do you recommend vaping Flavor (FLV) Sour Apple on its own? How much should I use?’* *‘Nic River PurNic (hit). I usually use Nic Rivers Smooth Salt but thought I’d try the Hit Salt.’*
	Logistics and shipping	Posts discussing vendors, supply availability, or shipping.	*‘Cheapest Army Post Office (APO) shipping for premix base?’* *‘Natural flavors? Best supplier?’*
	E-cigarette policy	Posts discussing policy changes, bans, or regulation-related shipping issues.	*‘Massachusetts will eliminate all options for DIY.’*
	Safety	Posts about safety of ingredients, methods, or potential harms.	*‘My mix has a purple hue. Any ideas what’s happening?’* *‘I just bought a batch of Nic Rivers Hit Salts (50/50 VG/PG mix) and it has a bright neon-yellow tint. Does this mean it’s gone bad?’*
	Advertisements	Posts advertising products or recruiting for research studies.	*‘University of Southern California (USC) is conducting a research study on new tobacco products. We seek healthy volunteers.’*

Experience was coded as: beginner, experienced, or NA, depending on whether users described themselves as new to mixing or demonstrated prior engagement with DIY practices, such as recipe development or vendor familiarity. This category was mutually exclusive. Post intent captured whether users were: asking questions (e.g. seeking flavor recommendations or troubleshooting help), or sharing experiences/offering advice (e.g. reviewing flavors, reflecting on their mixing process). Posts that did not fit clearly into either were marked NA. This category was also mutually exclusive. Post themes captured the main purpose of each post and were not mutually exclusive. Themes included: flavor profiles (e.g. posts discussing flavor notes, combinations, or reviews), recipes and ingredients (e.g. mixing instructions, ingredient sourcing, use of nicotine salts), safety (e.g. concerns about ingredient or method safety), e-cigarette policy (e.g. references to vaping laws or bans), logistics and shipping (e.g. vendor availability or delays), and advertisements (e.g. product promotions or study recruitment).

### Statistical analysis

Given the limited availability of literature pertaining to individuals that use DIY e-cigarette liquid methods, we applied principles of Grounded Theory to guide our content analysis^32^. Grounded Theory is best utilized when existing theories are insufficient to elaborate new concepts or when the importance of concepts has not been sufficiently explored for a given population^32^. In addition, we conducted descriptive statistical analyses to summarize coding categories and applied chi-squared (χ^2^) tests to compare distributions between the pre-ban and post-ban periods. All statistical analyses were performed using the analytical software R and Microsoft Excel with a two-sided significance threshold of p<0.05.

## RESULTS

A total of 5110 unique posts were extracted from the DIY_eJuice subreddit between 1 January 2019 and 2 January 2021. Of these, a stratified random subsample of 17% (n=800 posts) was selected for human coding, with an equal number of posts drawn from each period (400 pre-ban and 400 post-ban). These 800 posts were the analytic sample for the qualitative and statistical comparisons reported below.

### Current discourse of DIY in online communities

*Experience with DIY methods*

In the pre-ban period, 22.5% (n=90) of posts were classified as beginners, and 35.3% (n=141) as experienced DIY users, while 42.8% (n=171) could not be clearly categorized (Table 2). In the post-ban period, the proportion of beginners remained approximately constant at 22% (n=88), but the percentage of experienced users increased to 41.5% (n=166), reflecting an 18% relative increase.

**Table 2:** Descriptive statistics and thematic comparison of pre-ban and post-ban posts

*Category*	*Subcategory*	*Pre-Ban (N=400)*	*%*	*Post-Ban (N=400)*	*%*	*χ² (df, N)*	*p*
**Experience**	Beginner user	90	22.5	88	22.0	0.78 (1, 483)	0.38
	Experienced user	141	35.3	166	41.5	-	-
	Not classified (NA)	169	42.8	146	36.3	-	-
**Post intent**	Question/seeking advice	298	74.5	315	78.8	1.82 (1, 757)	0.18
	Sharing experience/ giving advice	79	19.8	65	16.3	-	-
	Not classified (NA)	23	5.8	20	5.0	-	-
**Themes**	Flavor profile	182	45.5	208	52.0	3.38 (1, 800)	0.08
	Recipes and ingredients	286	71.5	319	79.8	7.38 (1, 800)	0.007**
	Logistics and shipping	49	12.3	40	10.0	1.02 (1, 800)	0.36
	E-cigarette policy	14	3.5	33	8.3	8.10 (1, 800)	0.004**
	Safety	53	13.3	56	14.0	0.09 (1, 800)	0.80
	Advertisements	12	3.0	6	1.5	2.04 (1, 800)	0.23

Data source: DIY_eJuice subreddit posts (1 January 2019 – 2 January 2021), collected using Python Reddit API Wrapper (PRAW). Percentages are based on coded subsample of 800 posts (400 pre-ban, 400 post-ban). Statistical significance at p<0.05.

Thematic analysis revealed that beginner users frequently expressed appreciation and gratefulness for the DIY community and its learning environment. Several posts highlighted how helpful it was to observe others’ discussions and processes, particularly around flavor percentages and recipe decisions. Some users described placing their first orders or preparing to start mixing, often noting mistakes made during the ordering process. Others requested help identifying essential flavorings or creating specific clone recipes, such as strawberry coconut milk or menthol blends. A number of posts included questions about nicotine ratios, VG/PG blends, steeping, and flavor percentages, reflecting common early-stage confusion. Others described difficulties with taste or harshness in their mixes, attributing it to nicotine or incorrect ratios. Finally, many posts conveyed persistence and appreciation, with users stating their determination to continue learning and thanking others for their support.

In contrast, experienced users focused on complex discussions about flavor layering, fixing unbalanced mixes, and optimizing recipes through iterative testing. Many users demonstrated a high degree of method fluency, e.g. users were aware of assessing cooling agents, steeping times, and flavor volatilities in making liquids. Hardware choices and material interactions were also frequently discussed. Their posts often reflected a mindset of continuous experimentation, and interest in long-term recipe development. They also served as mentors within the community, offering advice, critiques, and resource guides to less experienced mixers.

*Intent*

Posts categorized were predominantly categorized as questions in both periods including 74.5% (n=298) in the pre-ban and increasing slightly to 78.8% (n=315) in the post-ban period. Meanwhile, posts classified as sharing personal experiences or advice slightly decreased from 19.8% (n=79) pre-ban to 16.3% (n=65) post-ban. These shifts were not statistically significant (χ^2^=1.82, p=0.18).

Thematic analysis revealed that users sharing personal experiences shared their own experiences with DIY mixing, from celebrating successful recipes and highlighting community-curated favorites to experimenting with techniques, lowering nicotine levels, and troubleshooting flavors. For example, every week a post entitled ‘recipe for the week’ shared a new mixing and flavor technique. On the other hand, the posts categorized as questions were about basic mixing processes, nicotine strength adjustments, steeping practices, and the effects of additives like sweeteners or cooling agents. Many users also sought troubleshooting help for issues such as flavor strength or harshness, issues with the coil etc. while others asked about recommended recipes, flavor brands, or storage practices.

*Other emerging themes*

Other posts included discussions on flavor profiles, DIY e-liquid recipes, safety of the methods, logistics and shipping, and changes in flavor policies. Discussions on recipes and ingredients increased from 71.5% (n=286) pre-ban to 79.8% (n=319) post-ban, whereas the discussion on flavor profiles increased from 45.5% (n=182) to 52% (n=208). The thematic content of the posts included users frequently asking for guidance on improving flavor experiences and including specific fruits like strawberry, mango, and watermelon or niche flavors like salted licorice or frozen lime. Other users shared or requested detailed recipes.

Similarly, policy-related discussions significantly increased from 3.5% (n=14) pre-ban to 8.3% (n=33). Analysis of policy-related discussion revealed that in 2019, policy-related posts largely reflected confusion and anxiety around newly announced flavor bans, with users questioning whether DIY supplies would remain legal, seeking ways to clone commercial juices, and voicing distrust of policymakers. By 2020, the discourse had shifted toward adaptation and long-term preparation, as users discussed stockpiling nicotine, safe storage for years, navigating new federal regulations [e.g. pre-market tobacco product applications (PMTA), vape mail bans], and identifying reliable vendors.

Posts about logistics (e.g. vendor and shipping issues) decreased slightly, from 12.3% (n=49) pre-ban to 10.0% (n=40) post-ban. In 2019, logistics discussions primarily focused on sales, reliable vendors, discontinued flavor lines, and shipping delays. In 2020, posts more often mentioned vendor closures, international sourcing, shipping restrictions, and PMTA-related updates.

Finally, the frequency of posts addressing safety concerns remained stable and relatively low (13.3% pre-ban vs 14.0% post-ban). The safety-related posts reveal widespread concerns about the quality, stability, and health effects of DIY e-liquids and nicotine products. Users often questioned whether bad smells, harshness, or odd flavors in nicotine and concentrates made them unsafe, and debated best practices for storage of e-cigarette liquids (fridge, freezer, or room temperature). Several posts specifically raised concerns about visible changes in the color or consistency of liquids, asking whether these alterations indicated spoilage or unsafe products. Others sought clarity on risks of ingredients (corn syrup, sugars, oils, alcohol, food flavorings) and whether certain additives or concentrates could cause harm or equipment damage (e.g. cracked tanks, harsh throat hit, inconsistent re-bottled flavors). Several users described adverse physical effects like coughing, reflux, or irritation, and asked if these were linked to nicotine strength, salts, or flavor compounds. Practical safety logistic including accurate nicotine measuring, cleaning bottles, and pod compatibility were also common.

### Effects of the 2020 flavor ban on the discourse on DIY e-cigarette communities

Posts discussing recipes and ingredients significantly increased from 71.5% (n=286) pre-ban to 79.8% (n=319) post-ban (χ^2^=7.38, p=0.007). Similarly, posts addressing e-cigarette policy significantly increased from 3.5% (n=14) pre-ban to 8.3% (n=33) post-ban (χ^2^=8.10, p=0.004).

Discussions on flavor profiles increased from 45.5% (n=182) pre-ban to 52.0% (n=208) post-ban, though this difference was not statistically significant (χ^2^=3.38, p=0.08). The proportion of posts classified as beginner users remained constant at 22.0% (n=88) across both periods (χ^2^=0.78, p=0.38), while the percentage of experienced users rose from 35.3% (n=141) pre-ban to 41.5% (n=166) postban. However, this increase was also not statistically significant.

Safety-related discussions showed minimal change, remaining at approximately 13.3% (n=53) pre-ban and 14.0% (n=56) post-ban (χ^2^=0.09, p=0.8). Posts related to logistics slightly decreased from 12.3% (n=49) pre-ban to 10.0% (n=40) post-ban, though not significantly (χ^2^=1.02, p=0.36). Lastly, advertisements decreased from 3.0% (n=12) pre-ban to 1.5% (n=6) post-ban, a change which was also not statistically significant (χ^2^=2.04, p=0.23).

## DISCUSSION

This study aims to describe and categorize the discourse of DIY e-cigarette liquid practice in online communities on DIY e-cigarette methods. The study further aimed to assess differences in the discourse before and after the Federal flavor ban on e-cigarettes in 2020. We found a significant increase in Reddit discussions related to DIY mixing methods, recipes, and ingredients following the FDA’s 2020 e-cigarette flavor ban. Additionally, there was a statistically significant rise in policy-related discussions. Conversely, discussions regarding safety did not show a statistically significant change between pre-ban and post-ban periods. Several findings from this study may warrant the attention of policymakers.

Firstly, in describing the experience of individuals that posted on Reddit, we found that the number of users coded as beginners was lower than the number of users coded as experienced in both the pre-ban and post-ban periods. Further, we also found that the number of experienced users increased in the post-ban period in frequency compared to the pre-ban period, though the increase was not statistically significant. Our findings align with the increase in e-cigarette use amongst young adults in the US between 2019 and 2021 from 8.8% in 2019 to 10.2% in 2021^33^. However, the absence of comprehensive data on the prevalence of DIY vaping methods makes it difficult to fully validate these results. Future studies should specifically focus on assessing the prevalence and characteristics of DIY e-cigarette practices or include questions regarding DIY practices in large-scale population surveys.

Secondly, we found that the majority of discussions, both in the form of questions and recommendations, on reddit were related primarily to methods for mixing and creating recipes. We also saw a significant increase in the discussions on recipes and other DIY methods in the post-ban period compared to the pre-ban period. Our findings are in-congruence with previous work on DIY e-cigarette liquid methods^18^, who found that over 65% used online forums to share and learn about new DIY mixing methods. Moreover, the significant increase in discussions in the post-ban period may possibly be due to the increase in the number of experienced users of the DIY methods.

Third, one of the more discussed topics in both pre- and post-ban periods were questions and recommendations on various DIY e-liquid flavor profiles. We noted that there was no change in the discussions related to flavor between the two periods. We believe that this finding is especially relevant in the post-ban period since the unchanged interest in flavors amongst the users may imply that the availability of flavors has not changed despite the ban due to availability of flavors through illicit channels^12,34^. Alternatively, it could also imply that the availability of disposable flavored e-cigarettes has led to a product substitution where individuals that previously used flavored e-cigarette pods with replaceable cartridges now use flavored e-cigarettes with disposable cartridges^35^.

Fourth, given that the DIY methods may be potentially harmful due to chemical constituents of commercially available ingredients to make e-liquids^18,36^, we also categorized discussions related to the safety of ingredients and methods in our dataset. We found that safety was only discussed in limited posts in the pre- and post-ban period. Further, most of the posts coded as safety discussions included questions about whether their liquids were safe for consumption due to changes in the color or consistency of liquids. The authors believe that further research is required to understand toxicity of DIY e-liquids and whether adverse outcomes have been reported from their consumption in clinical settings.

Fifth, logistics-related discussions were present in both study periods, though their focus shifted over time. In the pre-ban period, users primarily discussed practical concerns such as locating reliable vendors, taking advantage of sales, and managing discontinued flavor lines or routine shipping delays. In contrast, post-ban discussions more often referenced vendor closures, international sourcing difficulties, and shipping restrictions, as well as compliance challenges associated with PMTA regulations. While these posts represent a smaller proportion of the overall discourse compared to recipes or flavors, they highlight the ways in which broader regulatory and supply chain considerations shape access for DIY materials.

Finally, we found that there was a statistically significant change in the discussions related to the FDA 2020 ban in the post-ban period. However, the frequency of discussion related to policy changes was low in both pre-ban and post-ban period.

### Limitations

This study has several limitations. First, it relies only on data from the DIY_eJuice subreddit, which may not represent the entire population of DIY e-cigarette users, and have limited generatability. To address this limitation, we selected the largest and most active subreddit dedicated to DIY e-liquid discussions, which may increase the representativeness of the community.

Second, there is a possibility of misclassification during the coding process, which could influence thematic categorization. To address this limitation, we conducted multiple rounds of coder adjudication and aimed for high inter-rater reliability.

Third, the temporal scope of the study only captures information between 2019 and 2021, thereby missing long-term trends, and unmeasured factors, such as user characteristics, socio-cultural and geographical contexts, or contemporaneous events in the e-cigarette market. To address these issues, we strategically captured community responses immediately before and after the policy change to provide insight into short-term discourse shifts. However, we acknowledge that future epidemiological studies in the area may need to account for additional factors.

Fourth, this study is primarily descriptive and relies on proportions and chi-squared tests to compare themes across time periods, without conducting inferential or causal analysis. This limits the ability to estimate effect sizes, make population-level inferences, or establish causal relationships between the FDA flavor ban and changes in discourse. To address this limitation, we framed our findings as descriptive patterns; however, future work can apply more structured statistical and inferential approaches to test causal links more directly.

Fifth, lack of longitudinal data prevents tracking changes in individual behaviors over time. To address this, we utilized thematic coding and chi-squared analyses to compare group-level trends across distinct pre-ban and post-ban periods, providing meaningful comparative insights. Future research should incorporate longitudinal methods to track individual-level behaviors and explore emerging methods of nicotine and tobacco product use in broader surveillance efforts.

Finally, the study relies on self-reported content from Reddit users, which introduces the possibility of potential information bias. To overcome this bias, the authors suggest that future research can be conducted using other sources of health behavior assessment, such as surveys, to validate our findings

## CONCLUSIONS

This study provides a comprehensive analysis of the discourse in DIY e-cigarette communities on Reddit, highlighting the impact of the 2020 Federal flavor ban on these discussions. It reveals that while the number of experienced DIY users has increased, the overall discourse has shifted significantly towards discussions on mixing methods, recipes, and policy changes post-ban. Despite the marginal increase in safety-related discussions, these remain a smaller portion of the overall discourse, underscoring the need for further research into the safety and health impacts of DIY e-liquids. The findings suggest that regulatory changes influence the focus of online communities, but more data are needed to fully understand the implications for public health and safety.

## Data Availability

The data supporting this research were obtained from publicly available data from the social media platform, Reddit.
